# Design Space Exploration of a Multi-Model AI-Based Indoor Localization System

**DOI:** 10.3390/s22020570

**Published:** 2022-01-12

**Authors:** Konstantinos Kotrotsios, Anastasios Fanariotis, Helen-Catherine Leligou, Theofanis Orphanoudakis

**Affiliations:** School of Sciences and Technology, Hellenic Open University, 26334 Patras, Greece; afanariotis@eap.gr (A.F.); eleni.leligkou@ac.eap.gr (H.-C.L.); fanis@eap.gr (T.O.)

**Keywords:** indoor localization, Bluetooth, beacons, machine learning, embedded IPS

## Abstract

In this paper, we present the results of a performance evaluation and optimization process of an indoor positioning system (IPS) designed to operate on portable as well as miniaturized embedded systems. The proposed method uses the Received Signal Strength Indicator (RSSI) values from multiple Bluetooth Low-Energy (BLE) beacons scattered around interior spaces. The beacon signals were received from the user devices and processed through an RSSI filter and a group of machine learning (ML) models, in an arrangement of one model per detected node. Finally, a multilateration problem was solved using as an input the inferred distances from the advertising nodes and returning the final position approximation. In this work, we first presented the evaluation of different ML models for inferring the distance between the devices and the installed beacons by applying different optimization algorithms. Then, we presented model reduction methods to implement the optimized algorithm on the embedded system by appropriately adapting it to its constraint resources and compared the results, demonstrating the efficiency of the proposed method.

## 1. Introduction

Positioning systems and related services are gaining increased popularity, since they can assist users in a wide range of applications. The increased investments on installing the required infrastructure, the available technologies, and the performance of user devices that implement positioning solutions contribute to the increase of popularity. While there is a multitude of mature technologies available for outdoor positioning systems, offering accuracy down to the centimeter level [1], which cannot be said for indoor positioning systems (IPSs). The main reason for this is that almost every technology worth mentioning as mature and stable enough is based on a number (constellation) of satellites orbiting the planet, continuously transmitting positioning data [2]. For such systems to be functional, a clear line of sight towards the sky should be available at all times, something that is not possible for IPSs. Additionally, the complexity and energy requirements of user devices are constraints that limit their ranges of applications. The challenges of developing an IPS do not end there. Phenomena, such as signal multipathing, signal degradation, and line-of-sight loss that affect every non-inertial-based positioning system [3,4], start appearing, when the open space is confined by various obstacles. Outdoor environments, generally, present fewer obstacles that can reflect and degrade electromagnetic positioning signals even in populated urban areas, while indoor premises may contain obstacles such as walls, furniture, or in the worst case metallic appliances that reflect such signals.

In addition to the above considerations, IPS solutions should also take into account the fact that in their vast majority systems should address mobile applications and relevant requirements for execution on portable devices. This requirement turns the focus on technologies compatible with platforms and networked systems that operate in the framework of modern mobile communications and wireless sensor networks. However, most recent research efforts focus only on services provided directly to humans that use related devices for position tracking. The most prominent technology today in this context is based on 5G mobile communications [5] and the most widely deployed user devices designed to support 5G communications, generally named as smartphones (i.e., mobile phones usually supporting a common operating system such as Android and iOS and equipped with compatible subsystems and applications to support related services). With the advent of the Internet of things (IoT) and machine-to-machine (M2M) communications, IPS services can offer much more than human path-finding solutions. One such area is asset tracking, providing security (e.g., merchandise tracking), safety (e.g., construction/assembly line skipping avoidance), or complementing any IoT system that requires such services. This of course requires the application of IPSs on embedded, miniaturized, low-cost hardware, a requirement that is also researched in this paper.

The challenges presented for developing an IPS extend well beyond the area of technical feasibility. While outdoor positioning systems need extremely expensive satellites, their wide coverage justifies the extra cost since each constellation may cover and serve a large portion of the planet’s surface and population. On the other hand, each indoor area of a building must have a dedicated equivalent of a positioning constellation; thus, the costs of infrastructure equipment and its installation and maintenance are of great importance in such applications.

There is an extensive amount of research and methods for indoor positioning applications available nowadays, since there are different approaches that focus on different requirements. In general, the different proposed solutions to the internal positioning problem attempt to locate the position of a moving device with respect to some well-known points with fixed positions inside a two-dimensional space. In this work, we limit our discussion to two-dimensional spaces and exclude three-dimensional spaces, which is a generalized problem in the same category but more challenging. Thus, first, a way to infer the distance of a moving object from the fixed points in the interior space is needed. This can be achieved exploiting the relation between the distance of the transmitter and the fixed points inside the same space under an appropriate modelling of some measurable signal propagation characteristics between the two points. Different methods have been researched towards developing appropriate processing algorithms trying to filter out possible interference, noise, temporal variations in channel response, etc. or possibly exploit the fusion of data from multiple sources to improve the distance estimation. Once this is achieved, then a final step that falls within the general problem category of multilateration remains to be solved to determine the location of the movable device in space using multiple ranges (distances) between the device/point and multiple spatially separated known locations.

In this respect, a variety of methods have been researched for several years to circumvent both technical and economical challenges, such as ultra-wideband (UWB)-based technologies [6] that spread the positioning signal among a large range of frequencies with different propagation and multipathing behaviors, ultrasonic-based signaling [7] that eliminate the problems presented by electromagnetic signals, and more widely available Wi-Fi- or Bluetooth-based technologies [8,9] that are readily available and run complex digital signal-processing algorithms to increase the accuracy. There are of course others methods available such as radio-frequency identification (RFID) [10], which rely on the relatively dense distribution of transmitters in an area and return the position per transmitter on a sparse positioning grid. They, however, prove to be expensive because of the multitude of transmitters required, the excessive retrofitting effort, and the maintenance difficulty.

The most widely accepted method for IPS applications today is Received Signal Strength Indicator (RSSI) measurement over a Bluetooth or Wi-Fi group of signals [11,12]. Even if it is a well-known fact that the RSSI returns extremely unstable measurements, it is preferred since Wi-Fi and Bluetooth signals are already present in most buildings today. The development of the Bluetooth Low-Energy (BLE) protocol complementing Bluetooth protocol v4.0 or later presented the opportunity to developers to design more dense networks, increasing the accuracy, extensibility, and scalability of the systems while lowering the installation and maintenance costs because of the low-cost commercial solutions availability and the low-power functionality, making BLE/RSSI-based IPSs a top research subject.

Finally, as discussed above, there are numerous approaches towards developing an estimation based on the selected signal properties. In general, some forms of signal filtering and channel modelling have been attempted in order to establish this relation between the signal properties and the transmitter position including Kalman filtering, Bayesian fusion, dead reckoning [13], non-parametric classification such as k-Nearest Neighbors (k-NN)-based algorithms [14] or different combinations of the above. However, in recent years, the maturity of hardware and software platforms supporting neural networks (NNs) applying machine learning (ML) to a wide spectrum of problems including IPSs, has turned focus to the exploitation of ML-based approaches towards developing efficient position estimation methods [15,16].

Based on the above remarks, we initially investigated the application of ML techniques to infer the position of a BLE device moving within a fixed grid of an interior space covered by Bluetooth signals of beacons installed in fixed locations in [17]. In [18], an improved method was proposed to exploit the signals of different beacons in order to improve accuracy by developing independent ML models for each of the received RSSI signals to infer the distance of the device from the beacon and concurrently implementing a geometric approximation of the final position from these distance measurements. The improved method was shown to reduce the positioning error, while being independent of a preconfigured grid design of the interior space.

The goal of this paper is twofold. First, we investigated the relation of the NN complexity with the implementation accuracy and cost; then we researched the hypothesis that the development of an embedded system in the small form, capable of providing an acceptable accuracy level as an IPS is feasible. Therefore, we evaluated different ML methods and NN architectures and assessed the impact on the achievable accuracy of our proposed method as well as on the model size and related memory requirements to store the model and run the positioning application on the user device. Then, we explored state-of-the-art ML model optimization techniques to reduce the resulting model memory footprint by applying NN pruning and quantization. The resulting models were shown to finally achieve improved performance running on a microcontroller-based embedded device with a small-form factor.

Through the design space exploration in this work, a number of innovations were achieved. First, an outstanding indoor positioning accuracy was achieved through our optimized model in a typical application scenario under realistic conditions, reducing the average error below 25 cm. Additionally, a methodology towards the application performance enhancement through an ML model design was presented, demonstrating the interdependencies between the NN architectural parameters and the learning process to derive the final ML model to be executed by the application for the position inference. Our methodology was extended to the embedded system design space, applying the state-of-the-art TFLite Micro ML framework to port the ML models experimentally validated on smartphone devices to run on resource-constrained IoT nodes using as a reference development platform based on a state-of-the-art microcontroller with the BLE connectivity support. To our knowledge, it is the first time that NN pruning and quantization techniques to reduce the memory requirements down to sizes that could fit on the target device have been applied to an IPS solution, achieving this level of performance and experimentally validated.

Following the objectives described above, the rest of this paper is structured as follows: in Section 2, we reviewed relevant results published in the literature and discussed the contexts of their operation and performance limitations. In Section 3, we presented the details of the proposed method, the experimental setup, and the toolchain to derive the ML models, and in Section 4, we presented the results of a comparative performance evaluation of the different ML models we explored. In Section 5, we presented the process to port the proposed design on a resource-constrained embedded device and evaluated the results of applying NN pruning and quantization techniques to reduce the memory requirements down to sizes that could fit on the target device. Finally, in Section 6, we provided our concluding remarks.

## 2. Related Work and Requirement Analysis

Following the above approaches, the available technologies range from simple pressure sensors placed on the floor in a grid pattern, returning coarse-grained positioning information to extremely complex hybrid systems that use UWB signaling in combination with dead reckoning that provide a centimeter-level accuracy. In this section, typical technologies that have been most recently proposed were presented, briefly reviewing the state-of-the-art IPS ecosystem to put our work into context. For more extended and thorough reviews, the interested reader may see related work in [19,20,21,22].

IPSs that are based on UWB technologies: These are among those systems that provide the most accurate positioning information, even in less-than-ideal conditions such as in industrial environments [15]. Such systems use transmitters that signal the position information, and each tracking asset must bear a “tag” receiver that the transmitter recognizes and can communicate with it. Most of these systems employ a multi-model of time difference/time of arrival (TDoA/ToA) and angle of arrival (AoA) to allow trilateration for computing the position of each tag. To combine the information of TDoA and AoA and filter the noise, an extended Kalman filter (EKF) is applied to the measurements. While these systems provide excellent accuracy, they are considered high-cost solutions, are difficult to deploy, because of hardware requirements such as directional antennas capable of measuring AoA, and do not take advantage of any possibly already available IoT infrastructure [23]. They also work on a centralized mode where the position is calculated on either an anchor/transmitter or a remote server, increasing the single point of the failure probability.

Sparse-grid ultrasonic sensors IPSs: Such systems typically use one or multiple grids of ultrasonic transceivers, most of the time mounted on the ceiling of each area [24]. Each transceiver transmits a signal that can be received from a tag and may or may not have a payload of information about the transceiver’s location. At this point, there are two methods available to define the position information of the tag. The first way is that the tag answers the transceiver via ultrasonic signaling with its ID, and the information is pushed by the transceiver to the main server where the calculation of the tag’s position takes place. Alternatively, the tag itself communicates the information received by the transceiver directly to the server where the position information is calculated. While this type of system has a lower cost, it is rather difficult to deploy or retrofit to a building and extremely difficult to maintain since it is not as power-efficient as other technologies and requires constant power provisioning to the transceivers.

RSSI-based multilateration systems: These systems are commonly used in indoor low-accuracy positioning applications. They use signal strength measurements over a path-loss model to calculate the distance from each “visible” node and nowadays are considered trivial in their functionality. However, lately, new methods have emerged that may be described as “augmented” RSSI-based IPSs. These methods add some complexity to the basic path-loss RSSI multilateration models to bypass the well-known problems of the RSSI measurements instability. The main target of such systems is the profiling of the noise in an area and the application of a correction model that increases accuracy such as the division of an area into subspaces or zones and the application of a pre-established correction profile to measurements [25]. While these systems provide excellent results and are capable of functioning over existing infrastructure such as 2.4 GHz ZigBee transmitters, they are based on server–client models and are not device-agnostic. These requirements increase cost and deployment effort due to two reasons. Firstly, special software/firmware must be installed on the nodes, limiting the choice of nodes to those capable of supporting that firmware which may also impose the limitation that all nodes must be of the same type. Secondly at least one base station/concentrator must be added with a central server to process the final position calculations. Another similar method describes the mapping of an area as a multimap model adding ML-based correction to the model, giving again excellent results [26]. The nodes this time are BLE-based; thus, they are much more widely available but the system is also based on a server–client model, leading to a centralized position calculation that requires a server, while the tracked asset is an Android-based mobile phone, thus, there were no special power limitations in terms of energy consumption or computation power.

Obviously, all the above approaches have a merit in the quest for efficient internal positioning and originate from different starting points. The selection of a specific approach apart from the obvious requirement for the accurate and robust estimation of the device position additionally requires the examination of several dependencies and performance metrics that are tightly coupled with the final application domain and its requirements. In this work as already stated, we focused on mobile and embedded applications. Thus, we specifically focused on the following requirements that needed to be considered.

Existing infrastructure usage: Focusing on low-cost applications, the system should be able to use any existing deployed infrastructure inside a building where the device is expected to operate. This in turn is best served by RSSI-based systems using either existing Wi-Fi or BLE signaling for position inference, which are widely deployed in terms of infrastructure (access points or BLE devices scattered around the infrastructure) and supported by commercially available user devices.

In-node calculations: Position inference should be provided by the tracked node or asset. This means that the system should be able to calculate its position and not rely on remote/edge servers for computational offloading. This requirement simplifies the system deployment and maintenance while keeping costs to a minimum.

Low power capabilities: BLE is nowadays a widely accepted standard, when it comes to power-efficient commercial short-range/personal area network (PAN) communication applications. Other than this, the microcontroller should be able to support low-power modes that provide an opportunity to design or extend this system as a low-power low-maintenance device.

Surrounding devices agnostic: The system should rely on a common widespread protocol to measure the RSSI of omnipresent signals to guarantee extended and unlimited coverage. Thus, having identified BLE as the technology of choice, the system should be able to detect and measure RSSI values from BLE advertising devices. This is imposed as a requirement, since all BLE devices are capable to provide advertising even in the standby unconnected mode. Thus, it does not matter what kind of services each available BLE transmitter provides, as long as it can advertise its existence to the measuring node.

Transferable ML model: The trained model should be able to be transferred to a different device without the need for retraining while retaining its functionality within reasonable limits.

Considering the requirements and following the approach described above, in [17], a system for finding indoor locations with high precision in real time by making use of the Bluetooth RSSI values obtained from smartphones to infer the position of the user within a specific two-dimensional grid using ML was first described. An average positioning accuracy of around 70 cm and an inference with an error less than 1 m in 72% of the cases were achieved. In [18], an improved method was proposed to exploit the signals of all beacons within range. The enhanced method improves accuracy by developing independent ML models for each of the received RSSI signals to infer the distance of the device from the beacon and concurrently implementing a geometric approximation of the final position from these distance measurements. The improved method is shown to reduce the positioning error, while being independent of a preconfigured grid design of the interior space. Specifically, the results in [18] show that the system has an average accuracy of 69.58 cm and can predict the location with less than a meter accuracy in 80.55% of all cases and with an accuracy of less than 1.5 m in 93.92% of the cases.

As mentioned in the previous section, in this work, we extended the design proposed initially in [18] in two ways; first, we improved accuracy through an enhanced ML model design, investigating the relation between the model complexity, accuracy and cost; then, we applied a second-level optimization to achieve the porting of our implementation in an embedded system in the small form. In the following section, we described in more detail the proposed method and the process of the evaluation of different NN architectures. In subsequent sections, we explored state-of-the-art ML optimization techniques to reduce the resulting model memory footprint to fit on a microcontroller-based embedded device with a small-form factor and evaluate its performance.

## 3. Proposed System Design and Methodology

For reasons of completeness, we briefly described below the basic steps of the proposed approach originally presented in [18] to put the results of this work into context. We also described in this section the experimental setup and the hardware and software setup to derive the results of the evaluation of the enhanced models.

### 3.1. System Implementation and Experimentation

**Figure 1:**
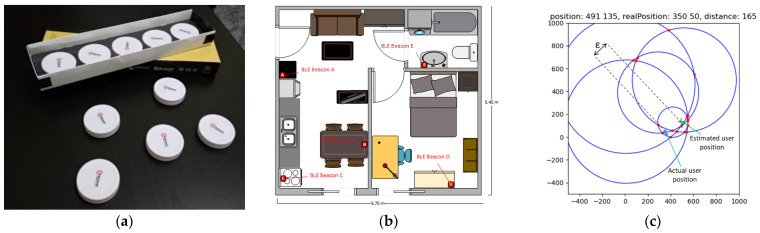
(**a**) Bluetooth beacons used in our experiments; (**b**) apartment map with beacon locations; (**c**) example result of the position inference method in [18].

Our internal location estimation operation was divided into three phases. First, our system made use of a set of ML models (one for each beacon within range). These models were used to infer the distance between the smartphone and each beacon based on the current RSSI-level measurement and its comparison against past measurements collected and used during the training phase. Second, the peripheries of circles that had as a center the beacon’ location and as a radius the inferred distance as well as their intersection points were calculated. Finally, the geometric median from the intersection points was calculated and was used as the estimated location (Figure 1c).

To evaluate our proposed algorithm through data collection in a real testbed, we used five iBKs105 Bluetooth beacons from Accent Systems (Figure 1a) that we placed in different locations around a 31 m^2^ apartment (Figure 1b). We selected a setup using one beacon per about 6 m^2^ of the internal space to achieve the best coverage with no blind spots scattered around the interior space in predefined and known positions (e.g., walls and ceiling). Then, by using an application that we developed on Android for a commercial smartphone (in our experiments a Xiaomi Mi A1 was used. Manufactured by Xiaomi and was purchased as a commercial device from a store in Thessaloniki, Greece), we collected measurements moving around the space of the apartment. For each measurement, we recorded the current position of the mobile, the RSSI values for all five beacons, the distance from each beacon, and a timestamp of the measurement.

### 3.2. ML Model Development

As mentioned above, our main objective in this work was to optimize the generation process of the ML models used to predict the distance between the mobile phone and the beacons, under a set of constraints that addresses several performance parameters. Thus, we evaluated the performance for a range of the basic NN architectural parameters that included the number of layers, number of neurons, and activation function. Our final objective was to improve the final inference accuracy, also considering and adjusting the learning rate and number of epochs as metrics related to the model convergence. To achieve this, we repeated the steps of the design and evaluation of a number of ML models, progressively rearranging the NN architecture and the learning process towards optimal ones for our ML model.

Before we presented the results of our design exploration and the optimization procedure, we listed below a list of configuration parameters of choice, common across all ML models and then we described the measurement and evaluation procedure we followed.

First, we normalized our data to make training more efficient. We used the Rectified Linear Unit (ReLU) activation function for all hidden layers. It is the default activation function for many types of neural networks, because a model that uses it is easier to train and often achieves better performance [27]. For the single-neuron output layer, we used the Linear Activation (LA) function. Another important task is the selection of the loss function. The mean squared error (MSE) or mean absolute error (MAE) are the two most common choices. As our goal was to minimize the average error of the distances between the device and the beacons, we chose the MAE, as it penalizes outliers and is easier to interpret.

The data that we used are a set of five RSSI values from five different beacons describing 11,110 measurements in total from the 31 m^2^ apartment shown in Figure 1b. Each measurement consisted of the respective 5 RSSI measurements, the actual distance from each beacon (five distances), a sequence number (ID), a point reference (point ID), and a timestamp. We collected the measurements from 110 predefined points with a distance between them of 50 cm, thus achieving an extensive coverage of the complete apartment area. During the data collection process, two persons on average were moving freely around the space, having normal activity (e.g., opening doors) to emulate representative everyday conditions. We split our dataset to 90% for training and 10% for validation. Therefore, for each one of our experiments, we used 9999 measurements for training and 1111 measurements for testing.

As mentioned above, the final position estimation is produced by the multilateration process implemented in the second and third phases of our method, but since this is based on the estimated distances from the different beacons, it is actually an ML-based inference that determines the overall accuracy of our method. Thus, it is of interest to explore the performance bounds of our design, evaluating potential ML model optimizations within the resource limitations of our reference implementation platform. To this end, we experimented through an evolutionary process with different ML models, deriving each time the appropriate parameters that contribute towards performance improvement as described in the following section.

For the development of the ML models, we used the TensorFlow framework [28,29] with Keras [30]. While TensorFlow is an infrastructure layer for differentiable programming, dealing with tensors, variables, and gradients, on top of it, we used Keras as a user interface for developing our deep learning models. Keras offers increased flexibility for dealing with layers, models, optimizers, loss functions, metrics, and more. It is an open-source software library that provides a Python interface for artificial neural networks that serves as the high-level application programming interface (API) for TensorFlow, simplifying the use of TensorFlow. Keras features consistent and simple APIs, and it can integrate deeply with low-level TensorFlow functionality, enabling the flexible configuration of workflows where any piece of functionality can be customized—a feature we exploited in our design space exploration.

## 4. ML Models Comparison

To begin our evaluation, we started with the development of the baseline network with two hidden layers having 64 neurons in both hidden layers used in [18] and chose RMSprop optimizer [31] with a default learning rate of 0.001 and trained for 1000 epochs. In Table 1 below, we present the results for all five beacons as well as the average MAE and MSE values across all five estimations. As observed in Table 1 with this configuration, we achieve an average MAE of 39.33 cm and an average MSE of 3214.83 cm.

**Figure 2:**
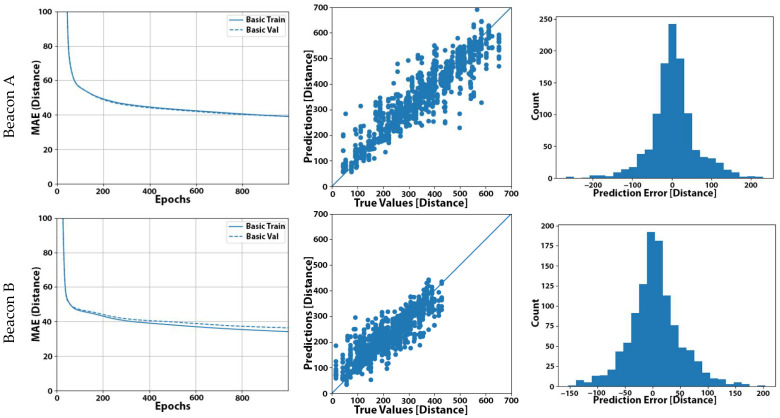

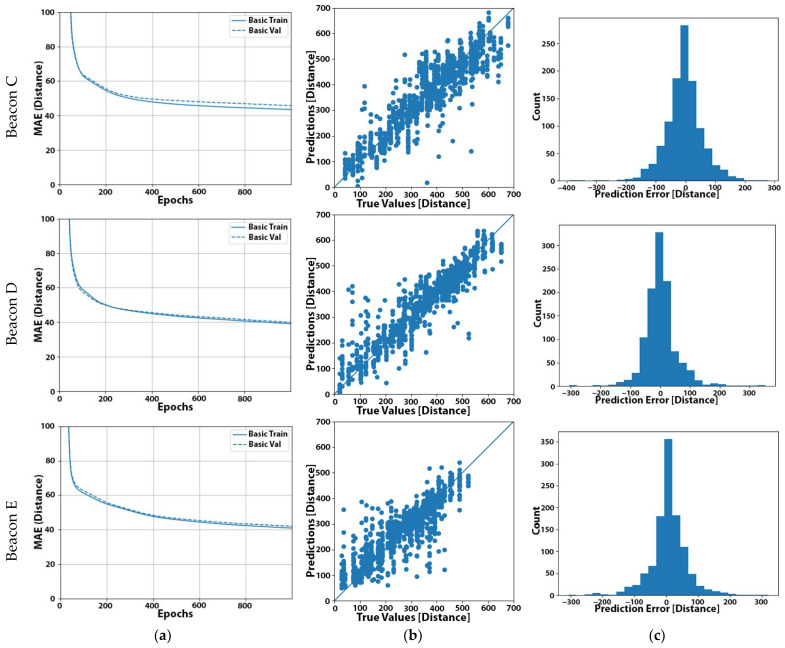
Experiment No. 1 results: (**a**) learning curve for our baseline model; (**b**) plot of the predicted distance (in cm) from our basic model against the actual distance of the user for the set of 1111 measurements; (**c**) distribution of the prediction error (in cm) using our basic model (distances expressed (in cm) in (**a**–**c**)).

One of our research questions is related to the convergence time of the learning process and the appropriate selection of epochs to let the system train in order to achieve good results. To answer this, in Figure 2a, we present the validation results related to the MAE convergence graphs for all beacons, and we can see that convergence was achieved at approximately 500 epochs. As we can observe for all five beacons, our models learnt very fast at the beginning of the process and started to converge after some epochs (300 to 500 epochs, depending on the model). This observation raised a reasonable doubt that the initial selection of 1000 epochs may not be optimal or even incur the negative effects of overfitting [32].

In Figure 2b, we present the scattering plots that described the deviation of the predicted distances from the actual distance of the user from the beacon for each measurement. Obviously, the closest the points reside to the diagonal line of the graph, the more accurate our model was. We also noted nearly equal positive and negative deviations contributed to a small total offset error. In Figure 2c, we present the distributions of the prediction error for all five beacons.

After inferring the distances from the five beacons, we ended up with the final device position estimation through the multilateration process described in the previous section. In Table 2 below, we show the values of the error distribution of the final device position estimation after the multilateration process for this initial configuration (based on the set of 1111 measurements that were used for testing the model estimation as before).

From the results of Table 2, we can conclude that with this baseline setup, our system achieved an average accuracy of 65.22 cm and can predict the location with less than a meter accuracy in 81.29% of all cases and with an accuracy of fewer than 1.5 m in 92.46% of the cases.

As a next step, we chose to test our hypothesis derived from our previous observations that the number of 1000 epochs for the learning process was overestimated. Thus, we conducted three experiments, keeping the same NN architecture, and repeated the learning process for the number of epochs to 500, 200, and 100 to see how this affected the predicted distance error using the resulting model. The results are displayed in Table 3. As we can observe, the selection of 500 epochs in experiment No. 1 yielded the minimum MAE. Actually, experiment No. 2 yielded even slightly better results in terms of the average MAE than with the baseline configuration, supporting our hypothesis for the negative impact of overfitting in the case of assigning excessive epochs to the learning process.

Having explored the impact of the number of epochs on performance bounds and in order to proceed with the validation of our learning process, we turned our focus on the optimization algorithm used for adjusting the rate of the learning process. Apart from the duration of the learning process and since we were interested in exploring deep learning models, we were also interested in evaluating the impact on the accuracy of the selected optimizations algorithm. Thus, in our next set of four experiments, we used the same NN architecture as in the previous experiments with two hidden layers with 64 neurons in both hidden layers, but we chose Adam algorithm for optimization and trainings for 1000, 500, 200, and 100 epochs. Adam optimization algorithm is an extension to stochastic gradient descent that has recently seen broader adoption for deep learning applications in computer vision and natural language processing [31]. As observed in Table 4, we obtained better results with the Adam optimizer than RMSprop, and a number of 500 epochs remained the choice, where the lowest average MAE was achieved.

To conclude our exploration of learning techniques in a different NN architectural configuration, we proceeded with the next four experiments, selecting an NN architecture with four hidden layers, the first two with 128 neurons and the next with 64 neurons. For the first two experiments, we used RMSprop, and for the other two, we used Adam optimizer to also conclude our optimizer benchmarking. We trained the models for 500 and 200 epochs that were shown to improve the accuracy in our previous experiments. The results are displayed in Table 5. From Table 5, we observed that the Adam optimizer still yielded better results in terms of the average MAE than RMSprop; thus, in the rest of our exploration, we used the Adam optimizer. On the other hand, the number of epochs did not have any significant impact on the results for the specific scenarios.

Starting with our previous observation that a deep neural network (DNN) configuration with an increased number of layers yielded better results, we proceeded to explore the bounds of this approach, experimenting with the following four DNN architectures by gradually increasing the number of neurons in each layer:

- Two hidden layers of 256 neurons, two hidden layers with 256 neurons, one hidden layer with 128 neurons, and one output layer (experiment No. 13);
- Two hidden layers of 512 neurons, two hidden layers with 256 neurons, one hidden layer with 128 neurons, and one output layer (experiment No. 14).

Then, the number of layers by adding one and two more hidden layers with 256 and 512 neurons, respectively, ending up with a final DNN with:

- Two hidden layers with 256 neurons, two hidden layers with 256 neurons, two hidden layers with 128 neurons, two hidden layers with 64 neurons, and one output layer (experiment No. 16);
- Two hidden layers with 512 neurons, two hidden layers with 256 neurons, two hidden layers with 128 neurons, two hidden layers with 64 neurons, and one output layer (experiment No. 16).

Adam algorithm remained the optimized algorithm, and we trained our model for 200 epochs in all cases. From the results in Table 6, we can observe that although the more complex DNN architectures yielded a decrease in the average MAE by more than 3 cm (compared to the results of Table 5), this was not significantly affected by the number of layers after one point. Only significant increases in both the number and the size of the layers (experiment No. 16) yielded an additional improvement in the accuracy of around 2 cm.

Since last experiment already resulted in a significantly larger model (requiring around 6 Mbytes of memory) and before continuing exploring more complex NN architectures, we noted that there still could be room for improvement without adding extra layers. Considering the fact that when working with several layers with ReLU activation we have a significant risk of dying neurons harming our performance [27], this in turn can lead to underfitting. Batch normalization (BN) is one of the best ways to handle this issue [33]. By applying the batch normalization process, we normalized the activation outputs of each layer for each batch to reduce the effect of extreme activations on the parameter training, which in turn reduced the risk of vanishing/exploding gradients. Motivated by this observation, in our next experiment, we applied batch normalization for each layer of the model developed in experiment No. 14, selected as a model using a modest-size NN with a good performance from the previous experiments. The result was the reduction of the error by about 3 cm compared with those of experiment No. 14, where we used the same architecture as shown in Table 7.

From the above encouraging observation, we can assume that adding the batch normalization contributed to bringing some of the neurons back to life, which increased our model results, changing underfitting to slight overfitting. At this point, as our next two experiments (18 and 19), we increased the number of the training epochs from 200 to 500 and 1000, in order to validate our previous conclusions regarding the training process in this case. The results are shown in Table 8.

Although the results in Table 8 validated our initial assumption about overfitting in the case of more complex DNN architectures, dropout has been proposed as a solution to DNN regularization and works with all types of network sizes and architectures [34]. Applying dropout randomly drops a portion of neurons in a layer in each epoch during training, which forces the remaining neurons to be more versatile. This decreases overfitting as one neuron can no longer map one specific instance, because it is not always there during training. To explore this possibility for improvement against the overfitting in the next two experiments (No. 20 and No. 21), we used dropout and trained our model for 1000 and 2000 epochs separately. The results are presented in Table 9, where we observed a small improvement in the accuracy with respect to the results of experiment No. 19. The DO(0.x) notation in the layers description column in Table 9 specifies what share of neurons we dropped, which translated into how much to regularize. As a default approach, we started with a dropout of around (0.3–0.5) in the largest layer and then reduced its rigidness in deeper layers [34]. The idea behind such an approach is that neurons in deeper networks tend to have more specific tasks and therefore dropping too many will increase the bias significantly.

**Figure 3:**
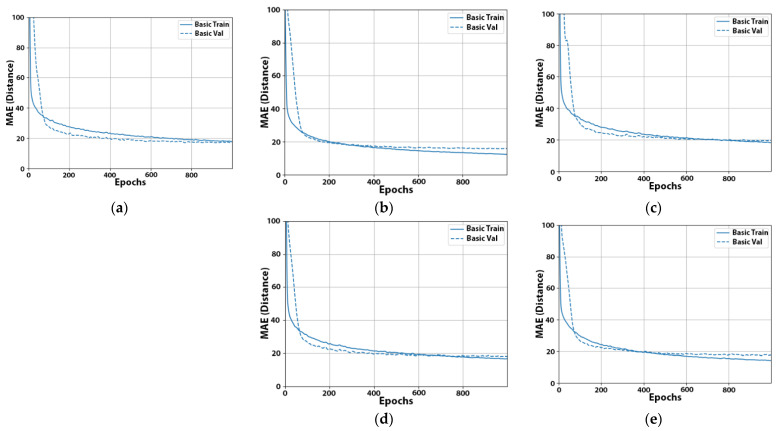
Learning curves of experiment No. 22 for beacons A (**a**), B (**b**), C (**c**), D (**d**), and E (**e**). Distances are expressed in cm.

Having achieved this through dropout, in our next experiment, we selected to reduce the learning rate. This would allow us to train our models faster at the beginning and then decrease the learning rate during later epochs to make the training more precise. In our case, the default Adam learning rate in Keras was 0.001, which was a bit high. Thus, we started with a learning rate of 0.005, and after 1000 epochs, the learning rate was finally decreased to 0.001. From the results in Table 10, we observed that tuning the learning rate helped us to finally improve the resulting validation error, while still keeping the learning curve healthy (Figure 3) without too much risk of overfitting.

As a final experiment, we selected to develop a larger network by adding one more hidden layer with 1024 neurons motivated by our previous observations that larger DNNs improved the accuracy in previous configurations. The new layer had the highest dropout rate (0.4), while the dropout was kept the same in the other layers. The results shown in Table 11 indeed demonstrated the improved accuracy.

At this point, we observed that the performance in terms of the MAE marginally improved compared to in simpler ML models (i.e., experiment No. 17, comparing the results of Table 11 with those of Table 7), while reaching the memory footprint limitations of our target device. Thus, it made no sense to continue further experimenting with alternative configurations.

**Figure 4:**
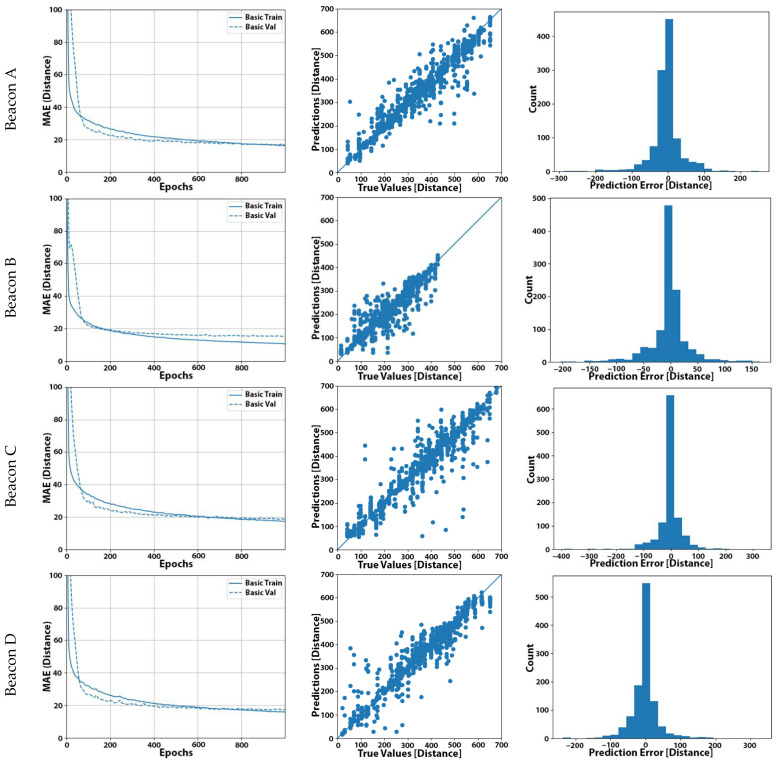

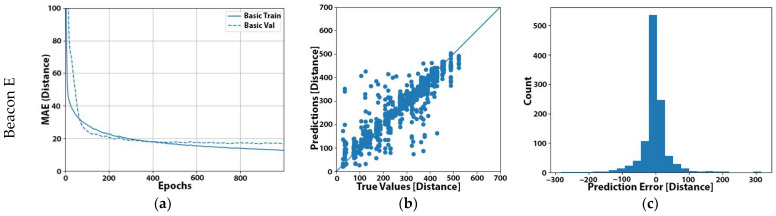
Experiment No. 23 results: (**a**) learning curve with the MAE of our final model; (**b**) plot of the predicted distance (in cm) from our final model against the actual distance of the user for the set of 1111 measurements; (**c**) distribution of the prediction error (in cm) using our final model. (Distances are expressed in cm in (**a**–**c**)).

For this last configuration that yielded the highest accuracy across all our experiments, we present, in Figure 4b, the plots that compared the final predicted distances as inferred by the five models against the actual distance of the device from each beacon. Comparing these plots with the respective plots in Figure 4c, we can observe how the measurement points were more densely spaced around the centerline and the improvement of the predicted distances.

**Figure 5:**
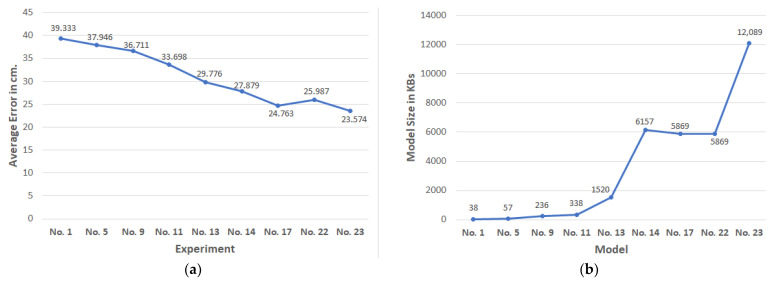
(**a**) Average error improvements for specific experiments; (**b**) models sizes for specific key models.

Finally, in Figure 5a, we present the average error improvements for the selected key experiments, and the resulting increases in models’ memory footprint are shown in Figure 5b.

Using the results of experiment No. 23 (Table 11), we fed them to the mulitlateration algorithm to predict the final location of the device and show, in Table 12, the values of the error distribution. The error calculation was based on the set of 1111 measurements that were used for testing the algorithm estimation against the actual user position. Thus, we can conclude that using the model of choice the proposed method can predict the user’s location with an average accuracy of 33.94 cm, i.e., an improvement of 31.28 cm on average compared to using the baseline model. Additionally, it can predict the location with a deviation of less than 50 cm in 73.84% of all cases and less than 1 m in 90.01% of the cases.

## 5. Porting to Resource-Constrained Devices

While modern smartphones have available computational and storage resources comparable to personal computers, the same does not apply to IoT devices. The initial Keras-based system with five models of 12 Mbytes each in a form of huge arrays is not efficient enough to run on such systems. Not only the limited storage presents a serious problem to be overcome, but also processing power is at least scarce, especially when the majority of these devices are based on a single- or dual-core processing unit without any additional offloading units, e.g., for networking functions and timer management.

### 5.1. Addressing the Constraints of Embedded Devices

The main method to address such constrains is to optimize the initial model in a way that is more suitable to run on an embedded device. The optimization includes various matrix conversions techniques with a main target of reducing complexity and size [35].

There are various tools that may be used to achieve this goal. However, only two of them support the creation of end-models that specifically target resource-constrained devices with enough support and stability to achieve stable experimentation results. This targeted sub-domain of ML today is called TinyML [36]. The first tool is TensorFlow Lite Micro, a Free and Open Source Software (FOSS) framework that recently has forked as a new development branch from the main TensorFlow Lite (TFLite) repository in order to allow developers to implement hardware-specific optimization methods [37]. The framework comes as a fully fledged framework that is partially compatible with TFLite and seamlessly connected with its full TensorFlow counterpart with software tools that enable the easy conversion between full TensorFlow or higher-level models such as Keras, TFLite, and TFLite Micro models.

The second tool is Pytorch GLOW AOT, a rather new framework described as “a machine learning compiler and execution engine for hardware accelerators” by its creators [38]. This is a rather new implementation in the TinyML ecosystem, and no official scientifically proven benchmarks exist. However, anecdotal benchmarks and comparisons [39] as well as the backing by big microcontroller producers such as NXP, ST Microelectronics, and other semiconductor design companies such as Intel and Synopsys makes this framework interesting.

Having said these, optimization can really get the implementation (size and latency-wise) up to a certain point and no further. In order to be successful on the final goal of designing a functional ML-based, small, embedded device, a suitable IoT system that offers “above the average capabilities” must be carefully selected. Of course, even the most powerful microcontrollers/SoCs today do not come anywhere near the computational power of modern CPUs, even those labeled as “power-efficient”.

Storage-wise (both for RAM and semi-permanent storage such as flash storage/HDDs) smartphones and personal computers stand at least three orders of magnitude above microcontrollers. The computational efficiency—without taking into account “instructions per cycle”—or the core count superiority of modern CPUs, microcontrollers are one order of magnitude slower. As a representative commercially available platform, we chose an SoC development device from the IoT ecosystem that offers both integrated support for wireless networking including BLE support, comes with a dual-core high-speed architecture and offers a variety of storage peripherals. This development board was based οn the ESP32 SoC with two Xtensa LX6 cores running at 240 MHz with 520 Kbytes SRAM, 448 Kbytes ROM, 4 Mbytes of flash memory, and another 4 Mbytes of PSRAM memory, with the last two connected as external peripheral devices. The device also had a secure digital (SD) card slot that was used as a permanent storage for the five ML models.

The minimum size of the TFLite model we achieved yielded five sub-models of 1 Mbyte each for a total size of 5 Mbytes following the process that was described in the following sub-section. The device supported a “flash scatter” scheme. Exploiting this scheme to fit the model to the device, all the PSRAM and a portion of the flash memory (1 MByte) were reserved, leaving 3 Mbytes of the storage for the rest of the application. The total size of the TFLite model had a size of 5 Mbytes in the form of five sub-models of 1 Mbyte each. Four of the TFLite sub-models were stored to an SD card and one worked as a constant array to the code, thus tagged by the compiler as data to be stored in the flash memory of the device. The device was set in a way that with each boot or reset it loaded the four sub-models from the SD card to its PSRAM. The fifth model was retained permanently in its flash memory. The total storage used was 4 Mbytes of PSRAM and 2.3 Mbytes of flash memory, bringing this application size to a total size of 6.2 Mbytes.

### 5.2. Model Optimization

Since TFLite Micro has today vastly greater support and maturity compared to every other TinyML framework, it was chosen as our implementation framework. Of course, in order to “shrink” the initial 60 Mbytes model into its final size of 5 Mbytes, the most optimal Keras-based model was optimized and converted into a TFLite model that was small enough to fit the target resource-constrained embedded device. The optimization procedure was divided into two steps. First, the model was pruned in order to increase its sparsity, making it easy to be re-encoded in a more efficient manner.

The target sparsity was set at a modest 0.8 factor, producing an 80% sparse model that was fine-tuned by retraining/re-validating for the same cycle/epoch times of the original model that was 1000 epochs. In order to reduce its size, the model was re-encoded from a classical X (m × n) arrays set to a TACO format [40], achieving a 3:1 reduction in size (12 Mbytes to 3 Mbytes per model).

The second step was to quantize the model in order to further reduce its size and its running efficiency. Quantization is the reduction of the model’s data types to a lower bit-width type [41]. The initial model was based on a float32 representation. Quantization converted it to an int8 data type, achieving a further 4:1 reduction in size and bringing it down to a little less than 1 Mbyte per model size for a total of 5 Mbytes for the whole inference system.

Furthermore, each calculation over the model’s stored values was made computationally cheaper, since operations can now be committed in the integer domain and require no “computationally expensive” floating point operations.

### 5.3. TensorFlow Lite Model Evaluation

**Figure 6:**
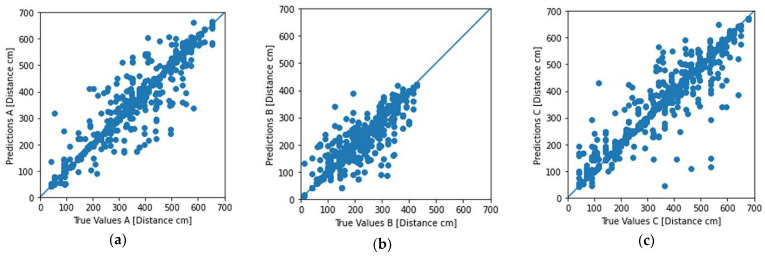

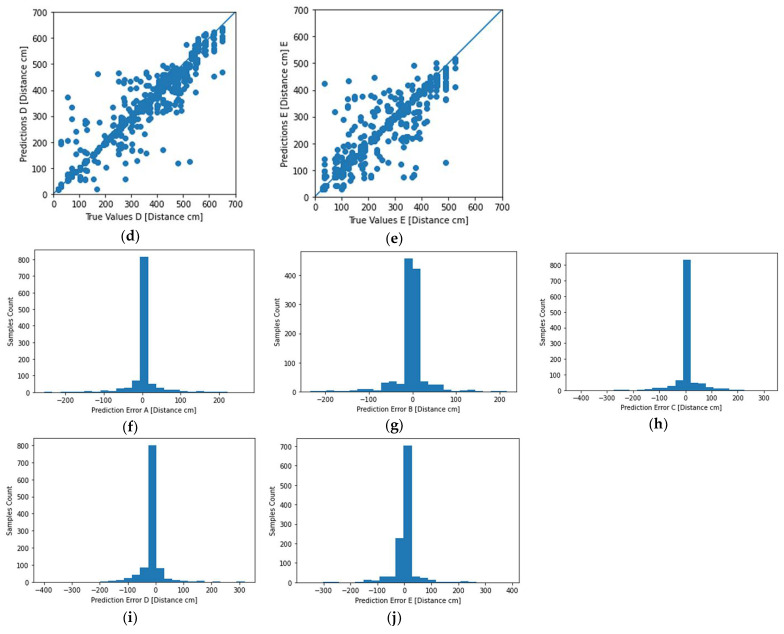
Inference scattering diagrams for nodes A (**a**), B (**b**), C (**c**), D (**d**), and E (**e**) (predicted distance in cm (y-axis) vs. true value (*x*-axis) in cm). Inference error distributions for nodes A (**f**), B (**g**), C (**h**), D (**i**), and E (**j**) (sample count (*y*-axis) vs. prediction error (*x*-axis) in cm).

The results for each beacon inference are depicted in Figure 6. The results included both the scatter diagram as well as a statistical error distribution for each inference made by the system.

A summary table of the optimization methods and the results obtained in each case are given in Table 13. It made it apparent here that indeed four out of five beacons were within 2 cm of the average inference offset from the real distance value with one beacon displaying a grater average offset of 11 cm.

While it is counter-intuitive to, more or less arbitrarily, remove information from a computational system and expect it to produce better results, this is not the case with DNN models. If performed correctly, a model rarely loses accuracy, and even in these cases, only a negligible percentage is lost. Most of the models keep their initial accuracies, and some of them show improvements [41,42].

The optimized TFLite model, in our case, showed accuracy improvements, bringing the average accuracy down to 19 cm. The MSE remained approximately the same, which further reinforced our measurement conclusions, since indeed the MSE is greatly affected by outliers. These outliers in our case were the samples with extreme levels of noise that remained statistically at the same levels for all the measurements.

While most ML models only need an absolute error indication as a metric of efficiency and correctness, in our case, we opted to extract the non-absolute average error as well. This gave us an application-specific (location vector) measurement for the beacon D, where an offset of –11 cm was apparent, a piece of information that may later be used in the final trilateration calculation as an offset correction to further improve the position accuracy.

## 6. Discussion and Conclusions

The continuous improvements in mobile communication networks and the smartphone device technology have turned the latter to an undisputed assistant to help mobile users to locate their positions and navigate in outdoor environments. At the same time, the prevailing BLE technology unveils new opportunities for developing new services including indoor positioning, which falls into the category of IoT applications. In a constantly changing technological environment, there is no “one-size-fits-all” solution, and different requirements and constraints must be dealt with appropriately. The maturity of ML model design and development frameworks have also opened new ways to exploit the power of modern embedded systems to implement ML-based solutions in different applications domains.

Building on the wide adoption of the smartphone as a multi-purpose platform and the power of ML as a method to derive results under uncertain external data inputs, we initially proposed a method for indoor localization based on ML to first infer the relevant device distances from multiple Bluetooth beacon points and then the actual position through multilateration. Starting with a baseline ML model configuration with two hidden layers with 64 neurons in both hidden layers, we showed that this baseline configuration selecting the RMSprop optimization algorithm with a default learning rate of 0.001 and trained using a training dataset of 9999 measurements for 1000 epochs yielded a mean MAE of 39.33 cm.

In this work, our main target was to increase the accuracy of the system through ML model optimization but at the same time explore the performance bounds, taking also the hardware platform limitations into account. We achieved this through a thorough design exploration process by implementing different optimization methods in a series of experiments. As the main optimization targets, we evaluated the learning process (in terms of epochs and rate), the optimization algorithm, and the NN architecture. We showed that Adam optimization yielded better results than RMSprop and a number of 200–500 epochs for training helped achieve convergence, avoiding overfitting for simpler models, but 1000 epochs may be required in the case of DNNs combined with dropout, batch normalization, and variable learning rates. There is clear evidence that larger-size DNNs yield improved accuracy at the cost of higher resource (mainly memory) requirements. Through our model optimization process, we succeeded in improving the average error of our system by 30.25 cm on average and achieving an accuracy of less than 1 m in 90.01% of the cases. This is achieved by the ML model using a DNN with one hidden layer with 1024 neurons, two hidden layers with 512 neurons, two hidden layers with 256 neurons, two hidden layers with 128 neurons, two hidden layers with 64 neurons, and one output layer, after applying dropout, batch normalization, optimization, and training, as shown in Table 11, requiring a 12 MBytes memory space for each model and 60 MBytes in total.

While the above IPS design was considered efficient considering a smartphone as a device of reference, we evaluated the possibility to achieve a similar performance in a resource-constrained device by selecting the ESP32-based module as a representative IoT device. We managed to achieve a 12:1 size reduction and observed an improvement of the model accuracy after pruning and quantization. As a future work, we will consider further analyzing how much inference latency is affected. It is a reasonable assumption that latency will improve since the model is smaller; thus, the array traversal throughout each inference calculation is faster. Furthermore, each element of the array is an integer, instead of a float, that eliminates the need for slow floating-point operations. Finally, the trade-off between the inference accuracy and the power consumption is also worth investigating.

## Figures and Tables

**Table 1:** Experiment No. 1 parameters and results.

#	Layers	Optimizer	Epochs	Model Size	Results per Beacon A/B/C/D/E—Average (in cm)
1	64, 64, 1	RMSprop (0.001)	1000	38,993 bytes	Mean absolute error (MAE): 40.17/34.51/45.52/37.18/39.29—**39.33** Mean squared error (MSE): 3332.66/2233.00/4010.05/3111.02/3387.43—**3214.83**

**Table 2:** Distance error distribution for our algorithm using the baseline model (in cm).

	0–25	25–50	50–75	75–100	100–125	125–150	>150
**Count**	226	327	221	121	84	49	83
**Percentage**	20.53	29.70	20.07	10.99	7.63	4.45	7.54

**Table 3:** Experiments No. 2, No. 3, and No. 4 parameters and results.

#	Layers	Optimizer	Epochs	Model Size	Results per Beacon A/B/C/D/E—Average (in cm)
2	64, 64, 1	RMSprop (0.001)	500	38,993 bytes	MAE: 40.09/ 33.12/ 42.23/ 39.51/ 38.97—**38.78** MSE: 3378.23/2071.59/3564.02/3404.97/3312.89—**3146.34**
3	64, 64, 1	RMSprop (0.001)	200	38,993 bytes	MAE: 39.59/33.91/43.72/38.12/42.34—**39.54** MSE: 3217.30/2164.52/3790.92/3199.90/3705.46—**3215.62**
4	64, 64, 1	RMSprop (0.001)	100	38,993 bytes	MAE: 42.25/36.04/46.28/41.70/44.71—**42.19** MSE: 3520.75/2380.55/4033.38/3616.56/3838.95—**3478.04**

**Table 4:** Experiments No. 5, No. 6, No. 7, and No. 8 parameters and results.

#	Layers	Optimizer	Epochs	Model Size	Results per Beacon A/B/C/D/E—Average (in cm)
5	64, 64, 1	Adam	1000	58,158 bytes	MAE: 37.73/33.56/43.09/35.93/39.42—**37.95** MSE: 3035.55/2143.05/3775.68/3034.54/3458.57—**3089.48**
6	64, 64, 1	Adam	500	58,158 bytes	MAE: 41.04/31.77/39.55/35.66/37.95—**37.19** MSE: 3450.60/1980.56/3350.01/2840.07/3274.71—**2979.19**
7	64, 64, 1	Adam	200	58,158 bytes	MAE: 38.44/32.67/43.02/36.87/41.75—**38.55** MSE: 3050.83/2048.23/3710.77/2981.14/3686.43—**3095.48**
8	64, 64, 1	Adam	100	58,158 bytes	MAE: 41.18/36.10/46.43/41.67/43.20—**41.71** MSE: 3507.90/2373.04/4011.43/3601.84/3756.70—**3450.18**

**Table 5:** Experiments No. 9, No. 10, No. 11, and No. 12 parameters and results.

#	Layers	Optimizer	Epochs	Model Size	Results per Beacon A/B/C/D/E—Average (in cm)
9	128, 128, 64, 64, 1	RMSprop (0.001)	500	241,273 bytes	MAE: 38.47/29.36/43.96/35.19/36.57—**36.71** MSE: 3111.98/1662.48/3749.46/2774.31/2983.28—**2856.30**
10	128, 128, 64, 64, 1	RMSprop (0.001)	200	241,273 bytes	MAE: 36.25/29.97/35.54/37.71/35.07—**34.91** MSE: 2802.38/1769.67/2780.90/3054.93/2853.39—**2652.25**
11	128, 128, 64, 64, 1	Adam	500	346,036 bytes	MAE: 36.54/27.01/35.82/35.52/33.60—**33.70** MSE: 2913.04/1570.19/2938.00/2867.50/2617.23—**2581.19**
12	128, 128, 64, 64, 1	Adam	200	346,036 bytes	MAE: 36.61/23.13/39.39/33.44/33.38—**33.19** MSE: 2809.78/1196.07/3349.89/2709.43/2676.73—**2548.38**

**Table 6:** Experiments No. 13, 14, 15, and 16 parameters and results.

#	Layers	Optimizer	Epochs	Model Size	Results per Beacon A/B/C/D/E—Average (in cm)
13	256, 256, 128, 128,64, 1	Adam	200	1,556,594 bytes	MAE: 31.35/22.56/35.93/27.65/32.27—**29.78** MSE: 2230.93/1252.20/3014.26/1965.78/2544.97—**2220.02**
14	512, 512, 256, 256, 128, 1	Adam	200	6,304,416 bytes	MAE: 27.72/20.13/37.34/28.04/28.14—**28.28** MSE: 1838.74/1117.00/2838.48/2058.70/2217.93—**2014.17**
15	256, 256, 128, 128, 64, 64, 1	Adam	200	5,955,675 bytes	MAE: 27.98/23.65/35.16/32.02/30.07—**29.78** MSE: 1970.75/1279.91/2849.43/2424.21/2575.79—**2220.02**
16	512, 512, 256, 256, 128, 128, 64, 64, 1	Adam	200	1,505,883 bytes	MAE: 28.26/21.02/32.89/28.13/29.10—**27.88** MSE: 1865.52/1162.03/2690.76/1982.32/2245.81—**1989.29**

**Table 7:** Experiment No. 17 parameters and results.

#	Layers	Optimizer	Epochs	Model Size	Results per Beacon A/B/C/D/E—Average (in cm)
17	512, BN, 512, BN, 256, BN, 256, BN, 128, 1	Adam	200	6,009,443 bytes	MAE: 25.65/20.47/28.82/23.35/25.52—**24.76** MSE: 1904.23/1251.65/2445.19/1631.84/2397.51—**1926.08**

**Table 8:** Experiments No. 18 and No. 19 parameters and results.

#	Layers	Optimizer	Epochs	Model Size	Results per Beacon A/B/C/D/E—Average (in cm)
18	512, BN, 512, BN, 256, BN, 256, BN, 128, 1	Adam	500	6,009,443 bytes	MAE: 26.35/22.73/29.91/23.83/29.97—**26.56** MSE: 1987.33/1400.96/2541.29/1856.24/2654.05—**2087.97**
19	512, BN, 512, BN, 256, BN, 256, BN, 128, 1	Adam	1000	6,009,443 bytes	MAE: 26.35/22.73/29.90/23.83/29.97—**26.56** MSE: 1987.33/1400.96/2541.29/1856.24/2654.05—**2087.98**

**Table 9:** Experiments No. 20 and No. 21 parameters and results.

#	Layers	Optimizer	Epochs	Model Size	Results per Beacon A/B/C/D/E—Average (in cm)
20	512, BN, DO(0.3), 512, BN, DO(0.3), 256, BN, DO(0.2), 256, BN, DO(0.2), 128, 1	Adam	1000	6,009,507 bytes	MAE: 28.19/21.46/30.86/26.49/24.95—**26.39** MSE: 2011.19/1266.64/2658.59/1882.59/2405.19—**2044.84**
21	512, BN, DO(0.3), 512, BN, DO(0.3), 256, BN, DO(0.2), 256, BN, DO(0.2), 128, 1	Adam	2000	6,009,507 bytes	MAE: 28.19/21.46/30.86/26.49/24.95—**26.39** MSE: 2011.19/1266.64/2658.59/1882.59/2405.19—**2044.84**

**Table 10:** Experiment No. 22 parameters and results.

#	Layers	Optimizer	Epochs	Model Size	Results per Beacon A/B/C/D/E—Average (in cm)
22	512, BN, DO(0.3), 512, BN, DO(0.3), 256, BN, DO(0.2), 256, BN, DO(0.2), 128, 1	Adam (lr = 0.005; decay = 5 × 10^–4^)	1000	6,009,587 bytes	MAE: 27.37/21.10/29.50/25.81/26.15—**25.99** MSE: 1921.92/1268.98/2522.69/1922.74/2439.31—**2015.12**

**Table 11:** Experiment No. 23 parameters and results.

#	Layers	Optimizer	Epochs	Model Size	Results per Beacon A/B/C/D/E—Average (in cm)
23	1024, BN, DO(0.4), 512, BN, DO(0.3), 512, BN, DO(0.3), 256, BN, DO(0.2), 256, BN, DO(0.2), 128, 1	Adam (lr = 0.005, decay = 5 × 10^−4^)	1000	12,378,812 bytes	MAE: 27.40/20.08/24.96/23.85/24.57—**23.57** MSE: 1820.57/1298.67/2429.45/1782.64/2271.46—**1920.56**

**Table 12:** Distance error (ε) distribution of the final model (in cm).

	0–25	25–50	50–75	75–100	100–125	125–150	>150
**Count**	768	119	65	48	37	29	45
**Percentage**	69.13	10.71	5.85	4.32	3.33	2.61	4.05

**Table 13:** TFLite optimization results.

	Optimization			Inference Results			
	Sparsity	Quantization	Fine-Tuning	Loss	MAE	MSE	Mean Error
Beacon A	0.8	float32 to int8	1000	18.4476	18.4476	1848.2050	0.6376
Beacon B	0.8	float32 to int8	1000	15.1017	15.1017	1194.3876	−2.2250
Beacon C	0.8	float32 to int8	1000	20.6127	20.6127	2342.6716	1.2697
Beacon D	0.8	float32 to int8	1000	23.8207	23.8207	2111.8499	−11.6955
Beacon E	0.8	float32 to int8	1000	18.0798	18.0798	2187.8782	−2.2230
**Total average**				**19.2125**	**19.2125**	**1936.9987**	**−2.8472**

## Data Availability

Data available in a publicly accessible repository that does not issue DOIs. Publicly available datasets were analyzed in this study. This data can be found here: https://github.com/kotrotskon/iBKs_regration.git (accessed on 15 December 2021).
